# HIV transmission in sub-Saharan Africa: excessive focus on heterosexual vaginal intercourse

**DOI:** 10.3389/fpubh.2025.1667141

**Published:** 2025-09-29

**Authors:** Sandro Vento

**Affiliations:** ^1^Department of Clinical Disciplines, Manash Kozybayev North Kazakhstan University, Petropavlovsk, Kazakhstan; ^2^Faculty of Medicine, University of Puthisastra, Phnom Penh, Cambodia

**Keywords:** HIV, HIV transmission, anal intercourse, men who have sex with men (MSM), heterosexual intercourse, HIV parenteral transmission, sub-Saharan Africa

## Introduction

Sub-Saharan Africa (SSA), and even more Southern Africa, has become the epicenter of the worldwide HIV epidemic since the late eighties-early nineties. In 2023, the average adult HIV prevalence was 15.3% in South Africa, Eswatini, Lesotho, Zimbabwe, Botswana, Mozambique, Namibia, Zambia, and Malawi, whereas it was only 1.45% in all the remaining countries in sub-Saharan Africa and just 0.4% in other low- and middle-income countries (1). The explanation for the high HIV prevalence in SSA and especially in the nine Southern Africa countries mentioned above, has long been transmission through heterosexual vaginal intercourse, mainly due to sexual promiscuity. The above explanation got widespread acceptance even though surveys about sexual behavior in 59 countries around the world reported a higher prevalence of pre-marital sex and of two or more sexual partners in the previous year in developed countries with much lower prevalence of HIV infections, compared with other countries, such as African ones (2).

Two recent papers have challenged the long-held assumption of a widespread transmission through heterosexual vaginal intercourse. In a scoping review of evidence from phylogenetic analyses of HIV-1 sequences, that examined sequences in samples collected in Botswana, Malawi, South Africa and Uganda, only 0.3%−7.5% of adult infections could be explained by household heterosexual intercourse (3). The review's authors outline that because many household partners refused to give blood or were not at home, were divorced or were dead, and phylogenetic analyses may have missed a few linked infections, percentages of infections from household sex partners may be higher. Nonetheless, it is clearly impossible to conclude that household heterosexual transmission is a major contributor to HIV epidemics in the studied communities (3).

In a data selection from 125 studies, the estimated pooled proportions of married men who have sex with men (MSM) were 4%, 8%, and 7% for Southern, East and West Africa, respectively (4). In Southern Africa, 29% of MSM had steady female partners, and in East Africa, 34% were currently or previously married to women (4). Reasons to marry women were a desire to have children, and to conform to heteronormative social norms and family pressure. Marriage was considered a way to stop same-sex behaviors or, conversely, a mean to secretly continue same-sex behaviors in a freer way (4). Hence, stable relationships with women are common in MSM in sub-Saharan Africa (SSA), where procreative wishes and a desire for secrecy often dissuade MSM from using HIV prevention methods with their female partners (4).

How do these two recent articles fit with previous evidence?

## Sexual transmission per coital event

In a 2009 systematic review, the pooled female-to-male (0.04% per act) and male-to-female (0.08%) estimates of the risk of HIV-1 transmission per heterosexual contact in high-income countries were lower than the low-income country female-to-male (0.38% per act) and male-to-female (0.30%) estimates (5). However, both were low rates, despite the greater heterogeneity of developing country estimates. The pooled receptive anal intercourse estimate was much higher (1.7% per act) (5). In a later systematic review, the estimated per-act HIV transmission risk per 100 exposures was greatest for blood transfusion (92.5), followed by mother-to-child transmission (22.6), receptive anal intercourse (1.38), needle-sharing injection drug use (0.63), and percutaneous needle stick injuries (0.23) (6). Risk for other sexual exposures were 0.04 for insertive penile–vaginal intercourse, 0.08 for receptive penile–vaginal intercourse, and 0.11 for insertive anal intercourse (6).

## HIV transmission in serodiscordant couples

The estimate from a study in Rakai, rural Uganda, was that fewer than one fifth of people with HIV infection had infected their home current heterosexual partners (7). As a comparison in a Western country, an old USA study involved 14 sexually active spouses of hemophilic men anti-HIV antibody positive for a mean of 46 ± 23 (SD) months (half of whom had had HIV antigenemia for a mean duration of 27 ± 23 months; six with AIDS). All couples were sexually active in a monogamous fashion, had been married for 13.5 ± 10.5 years and had had an average of four sexual intercourses per month (range: 1–12). None of the couples had used condoms prior to January 1986 (8). Antibodies to HIV were found in only one of the 14 wives, who had a long history of multiple sclerosis treated with immunosuppressant, and whose husband had AIDS (8), showing that heterosexual HIV transmission in serodiscordant couples is indeed an infrequent event.

## A relevant and overlooked role for heterosexual anal intercourse

In his 2019 manuscript, Michael A. Vance proposed a plausible explanation for the high prevalence of HIV infection in some SSA countries, that looks too high to be explained solely by transmission through heterosexual vaginal intercourse (9). He started with the well-known observation that in North America, Western Europe, and Australia HIV was largely spread through intravenous drug abuse with needle sharing, and anal intercourse in MSM. Vance stressed that the prevalence of anal intercourse among heterosexual Africans, being stigmatized, may also be underappreciated (10, 11) and that unprotected male–female anal intercourse could be more common than appreciated and could constitute an explanation more consistent with the observed sex ratios, even more so considering that MSM are often married to women (11). Importantly, a systematic review found that the HIV prevalence rate in SSA was 4.94 times higher among MSM than among men in the general population (12). Heterosexual anal sex is not at all rare in SSA. In a systematic review of published articles, prevalences of reported ever practicing anal intercourse ranged from 6.4 to 12.4% among adolescents, 0.3%−46.5% among university students and 4.3%−37.8% amongst combined population of adolescents and adults (13). Heterosexual oral and anal sexual acts are associated with some high-risk behaviors such as inconsistent condom use and multiple sexual partners (13).

Importantly, a recent mixed-methods systematic review found that in SSA, MSM perceive that the greatest risk of HIV acquisition comes from heterosexual/vaginal sex than from anal sex; in fact, they perceive lower or negligible HIV risk from sex with men, compared to sex with women (14). These misconceptions are driven by the predominant focus on heterosexual and vaginal HIV transmission in prevention information (14). Obviously, underestimation of the HIV acquisition risk through male anal intercourse poses major barriers to effective preventive behaviors and considerably increases the risk of transmission from MSM to their female sexual partners through anal intercourse (14).

In many SSA countries as well as in countries in other parts of the world, same-sex sexual acts are illegal and MSM experience strong social stigma, and feel pressured to have female partners. Therefore, as mentioned before, they could be a bridging group for HIV transmission to cisgender women. In a mixed-method, recent systematic review with meta-analyses of data of sex with women in MSM in sub-Saharan Africa, the pooled proportion of MSM who had sex with women was 58% (33%−83%) in East Africa in the previous 3 months, 27% (13%−48%) in Southern Africa and 50% (95% CI 39%−62%) in West Africa in the previous 6 months (15). 23% (16%−32%) of MSM in West Africa had condomless sex with a woman during the most recent encounter (15). Importantly, around one quarter of MSM had recent multiple female partners (15). HIV incidence remained high in MSM in Africa in 2020 (6.9 per 100 person-years, 95% CrI 3.1–27.6) and, impressively, 27–199 times higher than among all men (16). In the same year, just 73% (47–88) of all MSM living with HIV in Africa were estimated to be on ART, and only 69% (38–89) were estimated to be virally suppressed (16).

Female and male sex workers and people in prisons are intuitively at particularly high risk of acquiring/living with HIV. Data are not available for male sex workers. 2.5 million (95% uncertainty interval 1.9–3.1) female sex workers (FSWs) aged 15–49 years are estimated to live in sub-Saharan Africa, representing a 1.1% of all women of childbearing age (95% uncertainty interval 0.8%−1.3%) (17). HIV prevalence is particularly high in FSWs in Rwanda (51%) (18) and in Eswatini (70%) (19). Anal intercourse is common in FSWs (20–22) and often neither condoms nor lubricants are used by FSWs during it (19–21).

In a review of studies on HIV in sub-Saharan African prisons published between 2011 and 2015, the prevalence of HIV infection ranged from 2.3 to 34.9%, and prisoners almost constantly had a higher prevalence than did the non-incarcerated population in the same countries (23). Sexual violence, especially against young people, is not uncommon in prisons (24, 25) and exposure to forced penetrative sex was close to significance as a factor for HIV infection in Ghana's prisons (26). HIV suppression rates were found to be variable (94.25%−50%) in Cameroon (27).

## Non-sexual transmission of HIV

Another overlooked aspect of HIV spread that should be reconsidered is the role of parenteral transmission. Indirect suggestion for a role of parenteral transmission came from the findings of the “four African cities study,” where a high rate of partner change, sexual intercourse with prostitutes, concurrent sexual partnerships, large age difference between non-spousal partners, STDs, dry sex, and lack of condom use were not more common in the two high HIV prevalence cities (28), and from the much higher prevalence of HIV-1 among pregnant women in Zimbabwe vs. Tanzania (26 vs. 7%), despite a higher risky sexual behavior among Tanzanians (29). More direct evidence was provided by a study done at the University Teaching Hospital in Lusaka in 3,160 pregnant Zambian women, which found that medically administered intramuscular or intravenous injections in the previous 5 years were correlated with HIV prevalence, surpassing the contribution of sexual behaviors in a multivariable logistic regression analysis (30), and an investigation published in 2007 showed that failure to use autodisable syringes and greater tetanus coverage were strongly associated with greater HIV prevalence (31). Testing for HIV in discarded needles and syringe washes from people with HIV receiving injections in rural Cameroon in the early years of this millennium, allowed to amplify HIV-1 RNA from 34 of 103 intravenous injection syringes and 2/88 intramuscular injection syringes (32). In a Zimbabwean study of 1,807 adolescent females, 192 had HIV infection, 40.8% of whom had never had any sexual intercourse (33).

In the early years of the epidemics, rituals establishing “blood brotherhood,” ritual and medicinal enemas, ritual scarification, group circumcision were included among the possible transmission routes to be investigated (34). Years later, group scarification and the practice of blood oaths were thought to be risk factors (35) and in a study in rural Zimbabwe, scarification increased the risk of being infected with HIV (36). In a small case-control, prospective study done in 2007–2008 in a voluntary HIV testing clinic in Nigeria, seroconversion for HIV antibodies was associated with having shaved with a razor used previously by another individual and to have had surgery, blood transfusions, enemas, vaccinations, or infusions (37). In another case-control study in Kenya, HIV-infected children of uninfected mothers had had more blood testing, injections, infusions, and dental surgery by informal providers than their uninfected siblings (38). Data from the Kenya AIDS Indicator Survey 2012, done in participants aged 15–64 years, showed that men who had received ≥1 injection and women who had received an injection in the previous 12 months were significantly more likely to be HIV-infected (39).

Transmission through injections can be easier than generally assumed. A review of data on infective titer, viral load and injection inoculum volume concluded that the median transmission risks for unsafe intravenous or intramuscular injections using equipment cleaned but not sterilized after use on a pre-AIDS patient are 1.8 and 0.8%, respectively (40). However, the risk seems to be even higher if one examines the outbreaks related to contaminated medical equipment that occurred in different countries in Europe and Asia, in Libya, and in Mexico [reviewed in Ref. (41)]. Notably, less than 5% of documented cases had been reported from African countries.

Finally, the role of dental care and of barbers should be considered. Even though five patients of a dentist with AIDS were infected during their dental care in the USA (42), very surprisingly no such cases have been reported by any other country. Barbers' education on HIV prevention should be improved, as barbers in various countries (Ghana, Ethiopia, Nigeria, Morocco, Pakistan, and India) have inadequate knowledge and attitude and manifest poor prevention practices regarding HIV (43).

## Conclusions

WHO estimates that 650,000 people acquired HIV and 380,000 deaths occurred in the African region in 2024. To further diminish HIV incidence, exhaustive medical histories must be obtained by every individual found to have acquired the infection. If an outbreak, however small, occurs, it must be thoroughly investigated. Information about the risks of anal intercourse must be prioritized and integrated into sexual health programmes targeting the general population. The sterility of needles and other equipment used in medical settings and in rituals must be carefully and constantly controlled.

The emphasis on heterosexual transmission through vaginal intercourse and the neglect of transmission through anal intercourse must be re-evaluated also in other regions (nearly 113,000 HIV diagnoses were reported in 47 of 53 countries of the WHO European Region in 2023).

To effectively tackle the disproportioned burden of HIV in MSM, violence, stigma, discrimination and marginalization must be fought, and punitive and discriminatory norms abrogated (in 2020, MSM faced criminalization in 70 countries, including 32 in Africa) (44).
